# Biological compatibility between two temperate lineages of brown dog ticks, *Rhipicephalus sanguineus* (*sensu lato*)

**DOI:** 10.1186/s13071-018-2941-2

**Published:** 2018-07-09

**Authors:** Filipe Dantas-Torres, Maria Stefania Latrofa, Rafael Antonio Nascimento Ramos, Riccardo Paolo Lia, Gioia Capelli, Antonio Parisi, Daniele Porretta, Sandra Urbanelli, Domenico Otranto

**Affiliations:** 1 Department of Immunology, Aggeu Magalhães Institute, Oswaldo Cruz Foundation (Fiocruz), Recife, Pernambuco 50670420 Brazil; 2 Department of Veterinary Medicine, University of Bari, 70010 Valenzano, Bari, Italy; 3 Academic Unit of Garanhuns, Federal Rural University of Pernambuco, Garanhuns, Pernambuco 55292270 Brazil; 4 Istituto Zooprofilattico Sperimentale delle Venezie, 35020 Legnaro, Italy; 5 Istituto Zooprofilattico Sperimentale della Puglia e della Basilicata, Contrada S. Pietro Piturno, 70017 Putignano, Bari, Italy; 6 Department of Environmental Biology, Sapienza University of Rome, 00185 Rome, Italy

**Keywords:** Ticks, Genetics, Morphology, Biology, Crossbreeding, Paternal inheritance, Mitochondrial heteroplasmy

## Abstract

**Background:**

The brown dog tick *Rhipicephalus sanguineus* (*sensu stricto*) is reputed to be the most widespread tick of domestic dogs worldwide and has also been implicated in the transmission of many pathogens to dogs and humans. For more than two centuries, *Rh. sanguineus* (*s.s.*) was regarded as a single taxon, even considering its poor original description and the inexistence of a type specimen. However, genetic and crossbreeding experiments have indicated the existence of at least two distinct taxa within this name: the so-called “temperate” and “tropical” lineages of *Rh. sanguineus* (*sensu lato*). Recent genetic studies have also demonstrated the existence of additional lineages of *Rh. sanguineus* (*s.l.*) in Europe and Asia. Herein, we assessed the biological compatibility between two lineages of *Rh. sanguineus* (*s.l.*) found in southern Europe, namely *Rhipicephalus* sp. I (from Italy) and *Rhipicephalus* sp. II (from Portugal).

**Methods:**

Ticks morphologically identified as *Rh. sanguineus* (*s.l.*) were collected in southern Portugal and southern Italy. Tick colonies were established and crossbreeding experiments conducted. Morphological, biological and genetic analyses were conducted.

**Results:**

Crossbreeding experiments confirmed that ticks from the two studied lineages were able to mate and generate fertile hybrids. Hybrid adult ticks always presented the same genotype of the mother, confirming maternal inheritance of mtDNA. However, larvae and nymphs originated from *Rhipicephalus* sp. I females presented mtDNA genotype of either *Rhipicephalus* sp. I or *Rhipicephalus* sp. II, suggesting the occurrence of paternal inheritance or mitochondrial heteroplasmy. While biologically compatible, these lineages are distinct genetically and phenotypically.

**Conclusions:**

The temperate lineages of *Rh. sanguineus* (*s.l.*) studied herein are biologically compatible and genetic data obtained from both pure and hybrid lines indicate the occurrence of paternal inheritance or mitochondrial heteroplasmy. This study opens new research avenues and raises question regarding the usefulness of genetic data and crossbreeding experiments as criteria for the definition of cryptic species in ticks.

**Electronic supplementary material:**

The online version of this article (10.1186/s13071-018-2941-2) contains supplementary material, which is available to authorized users.

## Background

Ticks are external parasites of great medical and veterinary significance, causing incalculable losses to the livestock industry and a great burden on companion animals and human populations around the world [1, 2]. Climate changes, deforestation, biodiversity loss, animal and human population movements, changes in land-use, political and economic crises, among other factors, have induced changes in the distribution and epidemiological pattern of tick-borne diseases in various parts of the world [3].

Taxonomy and systematics of ticks have traditionally been based on morphological features. In the last three decades, the widespread use of genetic data and phylogenetic analysis has revolutionized both taxonomy and systematics of the Ixodida [4], but generated many questions as well about the specific identity of certain taxa [5, 6]. A classic example is what happened with the *Rhipicephalus sanguineus* group, which is an assembly of 17 morphologically similar tick species, including *Rh. sanguineus* (*sensu stricto*) [5, 6]. For over 200 years, *Rh. sanguineus* (*s.s.*) was believed to be a single taxon, even considering its poor original description and the inexistence of a type-specimen [5, 6]. However, it has been proposed that, until a neotype of *Rh. sanguineus* (*s.s.*) is designated, ticks assigned to this taxon should be referred to as *Rh. sanguineus* (*sensu lato*) [5, 6]. Indeed, genetic and crossbreeding experiments have indicated the existence of at least two distinct taxa within this name: the “temperate” and “tropical” lineages of *Rh. sanguineus* (*s.l.*) [7–18]. Additional genetic lineages have been identified in Europe and Asia, such as the lineage originally designated as “*Rhipicephalus* sp. I”, which is present in some temperate countries, such as Italy and Greece [13]. The presence of this lineage has also recently been confirmed in eastern European countries (e.g. Romania and Serbia) and in the Middle East (e.g. Israel) [19]. The existence of different lineages or cryptic species within *Rh. sanguineus* (*s.l.*) has implications, not only from a taxonomic perspective but also from a medico-veterinary standpoint. Indeed, ticks currently identified as *Rh. sanguineus* (*s.l.*) are vectors of various bacteria (e.g. *Rickettsia rickettsii*, *R. conorii* and *Ehrlichia canis*), protozoans (e.g. *Babesia vogeli* and *Hepatozoon canis*) causing diseases in dogs and/or humans [1, 5]. For instance, evidence indicates that the vector competence of the temperate and tropical lineages of *Rh. sanguineus* (*s.l.*) for *E. canis* may vary [20].

In Europe, at least two genetic lineages of *Rh. sanguineus* (*s.l.*) are known to occur: the so-called temperate lineage (also referred to as “*Rhipicephalus* sp. II”, a terminology that will be used herein for clarity’s sake, as we are dealing with two different temperate lineages) and *Rhipicephalus* sp. I [13, 19]. However, little is known about the current distribution (including areas of sympatry) of ticks belonging to these lineages and it is unknown whether they can breed and produce fertile hybrids in nature. Indeed, so far, only in Algeria and in southern Italy (Sicily insular region) ticks of both lineages have been retrieved [21]. The possible occurrence of incomplete reproductive isolation between the two lineages has been recently hypothesized based on the polymorphisms observed at the calreticulin gene (*crt* gene) [22]. In fact, ticks genetically assigned to *Rhipicephalus* sp. I and *Rhipicephalus* sp. II shared *crt* intron-present and intron-absent alleles and one *Rhipicephalus* sp. I individual from Putignano (Bari, southern Italy) showed both alleles, which could support the occurrence of a heterozygous genotype and ongoing gene flow. Alternatively, incomplete lineage sorting or past gene flow could explain the observed pattern at the *crt* gene locus. Within this context, the main objectives of this study were: (i) to characterize morphologically and molecularly the pure *Rhipicephalus* sp. I and *Rhipicephalus* sp. II tick lines; (ii) to verify the biological compatibility between ticks from these two lineages by performing crossbreeding experiments; and (iii) to assess the fertility of pure and hybrid tick lines.

## Methods

### Tick lines

Ticks used in this study originated from Portugal and Italy. In particular, engorged females genetically identified (see section “Genetic study”) as *Rhipicephalus* sp. I and *Rhipicephalus* sp. II were originally collected from sheltered dogs in Putignano (Bari, southern Italy) and privately-owned dogs living in Faro (southern Portugal), respectively. In the above-mentioned collection sites, only these genotypes have been found in previous studies [13, 19, 23].

Larvae (and subsequent nymphal and adult stages) originated from wild-caught, engorged females were defined as “wild type”. Ticks generated from males and females belonging to the same lineage were defined as “pure tick lines”, whereas ticks obtained by crossing different lineages were defined as “hybrid tick lines”. The first and second laboratory generations of crossed tick lines were designated as F_1_ and F_2_, respectively.

Throughout the study, all ticks were maintained in a laboratory incubator under controlled conditions of temperature, relative humidity and light, and fed on naïve rabbits, as described elsewhere [24].

### Morphological study

Unfed larvae and nymphs (10–20 days of age) from pure progenies were killed with warm water (50 °C) and placed in vials containing 70% ethanol. Then, they were mounted on glass slides using Hoyer’s solution [25] and examined under a light microscope. Newly emerged unfed adults from pure progenies were placed in vials containing 70% ethanol and examined directly under a stereomicroscope. All specimens were photographed and measurements taken using Leica Application Suite version 4.1 software (Leica Microsystems, Wetzlar, Germany). The following structures were measured: idiosoma length and width; scutum length and width; capitulum length; basis capituli length and width; hypostome length and palpal length; adanal plate length and width; adanal plate length/width ratio; dorsal prolongation of spiracular plate width; first festoon width; and the ratio between the width of the dorsal prolongation of spiracular plate and the width of the adjacent festoon (DPSP/AF ratio). The lengths of paired dorsal setae for larvae (scutal 3, central dorsal 1 and 2) and nymphs (central scutal 1 to 4) were also measured. Measurements are expressed as mean ± standard deviation and are provided in micrometres for larvae and in millimetres for nymphs and adults.

### Crossbreeding experiments

Crossbreeding experiments were carried out and the fertility of hybrid tick lines was assessed until the second generation (F_2_) (Table 1). The following parameters were analysed: female feeding period (days); female feeding success (%); engorgement weight (g); pre-oviposition period (days); oviposition period (days); engorged females laying eggs (%); egg-mass weight (g); blood meal conversion index (%); egg incubation period (days); egg hatchability (%); larval moulting success (%); nymphal moulting success (%); and sex ratio (female:male). The above parameters were also recorded for pure tick lines under the same conditions, being calculated as reported elsewhere [24].

**Table 1:** Tick groups used in this study

Group	Tick line	Specimens used
G1	Pure line of *Rhipicephalus* sp. II	10 females and 10 males from Portugal
G2	Pure line of *Rhipicephalus* sp. I	10 females and 10 males from Italy
G3	Crossed line with females of *Rhipicephalus* sp. II	10 females from Portugal and 10 males from Italy
G4	Crossed line with females of *Rhipicephalus* sp. I	10 females from Italy and 10 males from Portugal

Adult ticks used to establish both pure and crossed lines belonged to the wild type; they were obtained from nymphs that moulted from larvae obtained from wild-caught, engorged females. F_1_ and F_2_ generations from crossed lines are referred to as hybrids

### Genetic study

Wild type ticks belonging to the lineages *Rhipicephalus* sp. I and *Rhipicephalus* sp. II, as well as larvae, nymphs, males and females from laboratory pure and hybrid tick lines (G1, G2, G3 and G4), were used for genetic analysis. Genomic DNA was extracted from individual specimens using a commercial kit (DNeasy Blood & Tissue Kit, Qiagen GmbH, Hilden, Germany), following the manufacturer’s instructions. Partial cytochrome *c* oxidase subunit 1 (*cox*1) gene sequences (472 bp) were amplified using primers and PCR conditions described elsewhere [26]. Each reaction consisted of 4 μl of tick genomic DNA and 46 μl of PCR mix containing 2.5 mM MgCl_2_, 10 mM Tris-HCl (pH 8.3), and 50 mM KCl, 250 μM of each dNTP, 50 pmol of each primer and 1.25 U of AmpliTaq Gold (Applied Biosystems, Foster City, CA, USA). Approximately 100 ng of genomic DNA (with the exception of the no-template control) were added to each PCR. Amplified products were examined on 2% agarose gels stained with GelRed (VWR International PBI, Milan, Italy) and visualized on a GelLogic 100 gel documentation system (Kodak, New York, USA). Amplicons were purified and sequenced, in both directions using the same primers as for PCR, employing the Big Dye Terminator v.3.1 chemistry in an automated sequencer (3130 Genetic Analyzer, Applied Biosystems, Foster City, CA, USA). The *cox*1 gene sequences were aligned using the ClustalW program [27] and compared with those available in GenBank using the BLASTn tool (http://blast.ncbi.nlm.nih.gov/Blast.cgi).

### Statistical analysis

The mean differences of measurements were compared between F_1_ ticks (larvae, nymphs, males and females) of *Rhipicephalus* sp. I and *Rhipicephalus* sp. II, by analysis of variance (ANOVA). Morphometric data generated was also analysed through discriminant analysis to classify F_1_ ticks into different groups, based on a series of correlated variables (measurements). A structure matrix was generated for F_1_ larvae, nymphs, and adults (females and males) to highlight those variables that have the strongest correlations with the canonical function and that could help to discriminate between group 1 (G1) and group 2 (G2) (pure tick lines). The canonical function was then used to predict group membership and the success of assignment into the right group was expressed in percentage of correct classification. Statistical analysis was performed using SPSS for Windows, version 13.0.

## Results

### Morphometric study

Morphometric data obtained from F_1_ ticks belonging to G1 and G2 are provided in Tables 2, 3, 4, 5. Some variables showed cases of overlapping measurements, while others did not. Overall, the means of several measurements (7/10 for larvae, 6/10 for nymphs, 11/15 for males, 2/12 for females) were significantly different between G1 and G2 (Tables 2, 3, 4, 5). The discriminant analysis confirmed idiosoma width as the most discriminant variable to distinguish nymphs from G1 and G2, followed by scutum width and idiosoma length (Table 6). The discriminating power of the variables for larvae, males and females was lower than for nymphs (Table 6). Nonetheless, using discriminant analysis, 100% of the larvae and nymphs were correctly assigned to the original lineage (Table 7).

**Table 2:** Measurements (in μm) of and comparisons between F_1_ larvae from pure tick lines

Measurement	Group	Mean ± SD	Range	*F*	*P*
Idiosoma length	G1	577 ± 21	561–614	*F*_(1, 18)_ = 1.134	0.301
	G2	588 ± 22	552–620		
Idiosoma width	G1	396 ± 13	377–409	***F***_**(1, 18)**_ **= 17.255**	**0.001**
	G2	420 ± 13	402–441		
Scutum length	G1	207 ± 10	193–221	***F***_**(1, 18)**_ **= 7.816**	**0.012**
	G2	218 ± 6	208–230		
Scutum width	G1	324 ± 10	309–338	***F***_**(1, 18)**_ **= 8.634**	**0.009**
	G2	336 ± 8	326–353		
Dorsal setae length	G1	23 ± 1	22–25	***F***_**(1, 18)**_ **= 21.094**	**0.0001**
	G2	21 ± 1	19–23		
Capitulum length	G1	107 ± 10	97–129	***F***_**(1, 18)**_ **= 5.076**	**0.037**
	G2	116 ± 8	98–123		
Basis capituli length	G1	52 ± 4	46–59	*F*_**(**1, 18)_ = 0.020	0.890
	G2	52 ± 4	44–57		
Basis capituli width	G1	143 ± 6	133–154	***F***_**(1, 18)**_ **= 9.322**	**0.007**
	G2	150 ± 2	147–152		
Hypostome length	G1	55 ± 7	48–70	***F***_**(1, 18)**_ **= 9.732**	**0.006**
	G2	64 ± 5	54–71		
Palpal length	G1	79 ± 4	73–86	*F*_(1, 18)_ = 4.037	0.060
	G2	82 ± 3	75–86		

Statistically significant differences from ANOVA tests are indicated in bold

**Table 3:** Measurements (in mm) of and comparisons between F_1_ nymphs from pure tick lines

Measurements	Groups	Mean ± SD	Range	*F*	*P*
Idiosoma length	G1	1.40 ± 0.02	1.38–1.42	***F***_**(1, 18)**_ **= 34.165**	**< 0.00001**
	G2	1.34 ± 0.02	1.30–1.36		
Idiosoma width	G1	0.79 ± 0.02	0.76–0.83	***F***_**(1, 18)**_ **= 148.45**	**< 0.00001**
	G2	0.66 ± 0.03	0.64–0.71		
Scutum length	G1	0.53 ± 0.01	0.52–0.56	***F***_**(1, 18)**_ **= 11.650**	**0.003**
	G2	0.52 ± 0.01	0.51–0.53		
Scutum width	G1	0.60 ± 0.01	0.59–0.62	***F***_**(1, 18)**_ **= 59.163**	**< 0.00001**
	G2	0.57 ± 0.01	0.54–0.58		
Dorsal setae length	G1	0.26 ± 0.002	0.23–0.29	***F***_**(1, 18)**_ **= 29.215**	**< 0.00001**
	G2	0.21 ± 0.002	0.19–0.24		
Capitulum length	G1	0.23 ± 0.01	0.22–0.25	*F*_(1, 18)_ = 3.315	0.085
	G2	0.22 ± 0.01	0.21–0.24		
Basis capituli length	G1	0.12 ± 0.004	0.12–0.13	***F***_**(1, 18)**_ **= 4.765**	**0.043**
	G2	0.12 ± 0.01	0.10–0.13		
Basis capituli width	G1	0.34 ± 0.01	0.32–0.35	*F*_(1, 18)_ = 0.019	0.893
	G2	0.34 ± 0.01	0.32–0.34		
Hypostome length	G1	0.11 ± 0.01	0.99–0.12	*F*_(1, 18)_ = 0.023	0.880
	G2	0.11 ± 0.01	0.98–0.13		
Palpal length	G1	0.16 ± 0.01	0.14–0.17	*F*_(1, 18)_ = 0.139	0.713
	G2	0.16 ± 0.01	0.14–0.18		

Statistically significant differences from ANOVA tests are indicated in bold

**Table 4:** Measurements (in mm) of and comparisons between F_1_ males from pure tick lines

Measurements	Groups	Mean ± SD	Range	*F*	*P*
Idiosoma length	G1	3.33 ± 0.16	3.10–3.53	***F***_**(1, 18)**_ **= 7.039**	**0.016**
	G2	3.52 ± 0.17	3.23–3.75		
Idiosoma width	G1	1.72 ± 0.09	1.60–1.90	***F***_**(1, 18)**_ **= 7.039**	**< 0.00001**
	G2	1.92 ± 0.10	1.80–2.10		
Scutum length	G1	2.87 ± 0.11	2.73–3.02	*F*_(1, 18)_ = 2.572	0.126
	G2	2.96 ± 0.15	2.73–3.13		
Scutum width	G1	1.55 ± 0.07	1.41–1.65	***F***_**(1, 18)**_ **= 10.198**	**0.005**
	G2	1.70 ± 0.13	1.55–2.02		
Capitulum length	G1	0.50 ± 0.06	0.37–0.58	***F***_**(1, 18)**_ **= 11.066**	**0.004**
	G2	0.57 ± 0.04	0.52–0.61		
Basis capituli length	G1	0.27 ± 0.03	0.20–0.30	***F***_**(1, 18)**_ **= 7.000**	**0.016**
	G2	0.30 ± 0.02	0.30–0.30		
Basis capituli width	G1	0.72 ± 0.03	0.68–0.76	*F*_(1, 18)_ = 10.245	0.045
	G2	0.77 ± 0.04	0.70–0.82		
Hypostome length	G1	0.23 ± 0.06	0.08–0.28	*F*_(1, 18)_ = 4.607	0.046
	G2	0.27 ± 0.03	0.22–0.32		
Palpal length	G1	0.31 ± 0.02	0.28–0.35	*F*_(1, 18)_ = 0.352	0.560
	G2	0.31 ± 0.02	0.28–0.35		
Adanal plate length	G1	0.89 ± 0.07	0.79–1.00	*F*_(1, 18)_ = 0.472	0.501
	G2	0.92 ± 0.07	0.83–0.99		
Adanal plate width	G1	0.36 ± 0.04	0.30–0.43	***F***_**(1, 18)**_ **= 4.863**	**0.041**
	G2	0.39 ± 0.02	0.36–0.42		
Adanal plate length/width ratio	G1	2.51 ± 0.11	2.28–2.70	***F***_**(1, 18)**_ **= 8.920**	**0.008**
	G2	2.36 ± 0.11	2.20–2.61		
Dorsal prolongation of spiracular plate width	G1	0.07 ± 0.01	0.06–0.08	*F*_(1, 18)_ = 1.521	0.233
	G2	0.07 ± 0.01	0.06–0.09		
First festoon width	G1	0.13 ± 0.02	0.10–0.15	***F***_**(1, 18)**_ **= 21.550**	**< 0.00001**
	G2	0.16 ± 0.01	0.14–0.17		
DPSP/AF ratio^a^	G1	0.51 ± 0.08	0.45–0.63	***F***_**(1, 18)**_ **= 4.865**	**0.041**
	G2	0.45 ± 0.04	0.41–0.53		

^a^The ratio between the width dorsal prolongation of spiracular plate and the width of the adjacent festoon

Statistically significant differences from ANOVA tests are indicated in bold

**Table 5:** Measurements (in mm) of and comparisons between F_1_ females from pure tick lines

Measurements	Groups	Mean ± SD	Range	*F*	*P*
Idiosoma length	G1	3.27 ± 0.18	3.00–3.54	*F*_(1, 18)_ = 0.022	0.885
	G2	3.26 ± 0.14	2.92–3.42		
Idiosoma width	G1	1.56 ± 0.08	1.50–1.70	*F*_(1, 18)_ = 1.694	0.210
	G2	1.60 ± 0.07	1.50–1.70		
Scutum length	G1	1.57 ± 0.07	1.44–1.64	***F***_**(1, 18)**_ **= 7.704**	**0.012**
	G2	1.63 ± 0.04	1.58–1.70		
Scutum width	G1	1.37 ± 0.08	1.27–1.51	*F*_(1, 18)_ = 1.662	0.214
	G2	1.41 ± 0.04	1.37–1.48		
Capitulum length	G1	0.62 ± 0.04	0.57–0.67	*F*_(1, 18)_ = 0.890	0.358
	G2	0.64 ± 0.04	0.58–0.69		
Basis capituli length	G1	0.30 ± 0.03	0.30–0.40	*F*_(1, 18)_ = 0.859	0.366
	G2	0.30 ± 0.01	0.30–0.30		
Basis capituli width	G1	0.82 ± 0.02	0.78–0.84	***F***_**(1, 18)**_ **= 5.468**	**0.031**
	G2	0.84 ± 0.02	0.79–0.86		
Hypostome length	G1	0.32 ± 0.02	0.29–0.36	*F*_(1, 18)_ = 3.115	0.095
	G2	0.34 ± 0.04	0.28–0.39		
Palpal length	G1	0.38 ± 0.02	0.35–0.40	*F*_(1, 18)_ = 0.692	0.416
	G2	0.37 ± 0.01	0.35–0.38		
Dorsal prolongation of spiracular plate width	G1	0.07 ± 0.01	0.06–0.08	*F*_(1, 18)_ = 0.367	0.552
	G2	0.07 ± 0.01	0.06–0.08		
First festoon width	G1	0.18 ± 0.02	0.15–0.21	*F*_(1, 18)_ = 1.429	0.247
	G2	0.17 ± 0.01	0.15–0.18		

Statistically significant differences from ANOVA tests are indicated in bold

**Table 6:** Pooled within-groups correlations between pure lines ticks (G1 and G2), discriminating variables and standardized canonical discriminant functions

Variable	Absolute size of correlation within function			
	Larvae	Nymphs	Females	Males
Dorsal setae length	**-0.413**	0.276	–	–
Idiosoma width	**0.374**	**0.622**	0.165	**0.314**
Hypostome length	**0.281**	0.008	0.224	0.137
Basis capituli width	0.275	-0.007	**0.296**	0.204
Scutum width	0.264	**0.393**	0.163	0.204
Scutum length	0.251	0.174	**0.352**	0.102
Capitulum length	0.203	0.093	0.120	0.212
Palpal length	0.181	0.019	-0.105	0.038
Idiosoma length	0.096	**0.298**	-0.019	0.169
Basis capituli length	0.013	0.111	-0.117	0.169
DPSP/AF ratio^a^	–	–	0.179	-0.141
First festoon width	–	–	-0.151	**0.296**
Dorsal prolongation of spiracular plate width	–	–	0.077	0.079
Adanal plate length	–	–	–	0.044
Adanal plate width	–	–	–	0.141
Adanal plate length/width ratio	–	–	–	-0.206

^a^The ratio between the width dorsal prolongation of spiracular plate and the width of the adjacent festoon

Bold indicates the higher correlation within function for each tick developmental stage

**Table 7:** Classification of F_1_ tick specimens as belonging to G1 or G2 based on discriminant analysis

Group of origin	Predicted group membership							
	Larvae		Nymphs		Females		Males	
	G1	G2	G1	G2	G1	G2	G1	G2
G1	10	0	10	0	7	3	9	1
G2	0	10	0	10	3	7	2	8
Correctly classified (%)^a^	100		100		70		85	

^a^Percentage of ticks correctly classified as belonging to a particular group

### Crossbreeding experiments

Crossbreeding experiments showed that *Rhipicephalus* sp. I males were able to mate with *Rhipicephalus* sp. II females, and *vice versa*, generating fertile hybrids. Detailed data from biological parameters recorded for pure and hybrid tick lines (G3 and G4) are provided in Table 8. Engorged F_1_ and F_2_ females from all groups showed similar patterns in terms of feeding success, engorgement weight, pre-oviposition period, oviposition period, egg-mass weight produced and blood meal conversion index. However, with regard to hybrids, engorged F_2_ females were heavier than those of F_1_, although they did not produce greater egg masses. Indeed, they presented lower blood meal conversion index as compared with F_1_ females. The minimum egg incubation period and egg hatchability were also similar across generations (Table 8). No noticeable differences were found in relation to larval and nymphs moulting rates, with the exception of the lowest moulting rates recorded for F_2_ larvae (80%) and nymphs (95.3%) from the hybrid line with females of *Rhipicephalus* sp. I (Table 8). No parthenogenesis was observed in any of the groups; the proportion of males in F_1_ ranged between 45–50%, with sex ratios (females:males) close to unity in all groups (1:1 in G1, G2 and G4, and 1:0.8 in G3).

**Table 8:** Biological parameters recorded for different tick lines^a^ used in this study

Parameters	Pure lines		Crossed lines		Hybrid lines (F_1_)		Hybrid lines (F_2_)	
	G1	G2	G3	G4	G3	G4	G3	G4
Female feeding period (days)	18.5 ± 1.4	21.4 ± 1.2	14.6 ± 2.3	18.0 ± 0.0	16.0 ± 0.0	13.3 ± 2.00	12.0 ± 0.0	11.0 ± 0.0
Female feeding success (%)	40.0	55.0	30.0	50.0	50.0	60.0	60.0	40.0
Engorgement weight (g)	0.2 ± 45.6	0.3 ± 45.4	0.3 ± 15.4	0.3 ± 45.8	0.3 ± 46.4	0.3 ± 88.1	0.3 ± 0.02	0.4 ± 0.0
Pre-oviposition period (days)	3.3 ± 0.7	3.0 ± 1.0	2.1 ± 1.5	2.2 ± 1.3	3.4 ± 1.2	3.0 ± 0.6	1.0 ± 0.0	1.0 ± 0.0
Oviposition period (days)	13.3 ± 1.0	14.2 ± 1.9	16.6 ± 2.0	15.4 ± 1.5	14.2 ± 2.6	17.3 ± 2.3	14.2 ± 2.9	11.3 ± 0.5
Engorged females laying eggs (%)	100.0	100.0	100.0	100.0	100.0	100.0	100.0	87.5
Egg-mass weight (g)	0.1 ± 33.5	0.2 ± 29.4	0.2 ± 15.2	0.2 ± 40.5	0.2 ± 0.1	0.2 ± 0.1	0.2 ± 30.3	0.2 ± 21.0
Blood meal conversion index (%)	57.4	66.2	67.1	68.4	73.4	78.2	59.0	66.9
Egg incubation period (days)	5.5 ± 0.5	6.4 ± 0.9	5.5 ± 0.5	7.9 ± 1.1	9.2 ± 2.2	12.3 ± 2.3	10.5 ± 1.1	12.5 ± 1.8
Egg hatchability (%)	100.0	100.0	95.5	99.4	86.0	93.0	100.0	98.0
Larval moulting success (%)	99.6	98.8	98.6	98.7	99.5	99.5	95.0	80.0
Nymphal moulting success (%)	100.0	100.0	100.0	100.0	96.3	99.5	98.2	95.3

^a^Crossed lines refer to pure females from a given lineage that mated with pure males from a different lineage. Larvae and nymphs from these crosses are hybrids generated from these crosses. Hybrid lines refer to hybrid males and females (and their offspring), obtained from crossed tick lines

### Genetic identification and mitochondrial DNA inheritance

In total, 122 partial *cox*1 sequences were generated and analysed [Additional files 1 and 2]. Sequences obtained from “wild-type” ticks shared 99–100% nucleotide identity with sequences for reference strains of *Rhipicephalus* sp. I (GenBank: KC243884, KC243883) or *Rhipicephalus* sp. II (GenBank: KC243891) retrieved from GenBank, confirming the genetic identity of the ticks used in this study. No ambiguous single nucleotide polymorphisms were detected for the sequence obtained from G1 and G2 offspring specimens.

All immature and adult F_1_ ticks from pure and hybrid lines showed the maternal mtDNA as expected, with the exception of larvae and nymphs originating from *Rhipicephalus* sp. I females, which showed either the *Rhipicephalus* sp. I or *Rhipicephalus* sp. II genotype. A high percentage of nucleotide identity (99–100%) was recorded by comparing all F_1_ tick sequences with the reference strains, for each group and developmental stage examined.

## Discussion

In the present study, we conducted morphometric, biological and genetic comparisons between two temperate lineages of *Rh. sanguineus* (*s.l.*), namely *Rhipicephalus* sp. I and *Rhipicephalus* sp. II. Phenotypically, these lineages are very similar, but morphometric analysis revealed differences for some measurements, especially for larvae and nymphs (Table 6). In fact, all larvae and nymphs were correctly classified by discriminant analysis (Table 7). Scutal and alloscutal setae, along with idiosoma width, scutum width and length were among the best discriminating variables for larvae and nymphs of *Rhipicephalus* sp. I and *Rhipicephalus* sp. II. As a matter of fact, some of these characters (e.g. scutal and alloscutal setae) had already been suggested as reliable morphological characters for separating *Rh. sanguineus* (*s.l.*) and *R. turanicus* [28]. Altogether, our results indicate that the combined analysis of several measurements is the most reliable way to separate morphologically larvae and nymphs of these lineages.

Previous studies using ticks belonging to the tropical and temperate lineages of *Rh. sanguineus* (*s.l.*) revealed that these ticks could mate and generate viable hybrids [11, 29]. Most of the eggs produced by hybrid females obtained in these studies were infertile, but some larvae successfully hatched in at least one study [29]. This indicates that the tropical and temperate lineages of *Rh. sanguineus* (*s.l.*) have been separated for quite some time; this hypothesis is also supported by the differences found in their mitochondrial genomes [14]. A recent laboratory study suggested that their geographical isolation may have been driven by climatic factors [30].

Our experiments confirmed that *Rhipicephalus* sp. I males were able to mate with *Rhipicephalus* sp. II females, and *vice versa*, generating fertile hybrids. While this may suggest that these lineages are conspecific, previous studies have shown hybridization to be possible in some tick species, under both laboratory [31, 32] and natural conditions [33]. Therefore, the ability to mate and generate fertile descendants cannot be used as a sole criterion to assess conspecificity.

It is worth nothing that, while morphologically similar and biologically compatible, *Rhipicephalus* sp. I and *Rhipicephalus* sp. II are genetically quite divergent, i.e. up to 7, 10.4 and 12.5% for *16S* rRNA, *12S* rRNA and *cox*1 genes, respectively [13]. To put this into perspective, the pairwise distances (for *cox*1 sequences) between *Rhipicephalus* sp. I and *Rh. guilhoni*, *Rh. pusillus*, *Rh. turanicus*, and tropical lineage of *Rh. sanguineus* (*s.l.*) were 10%, 11.1%, 11.7% and 12.3%, respectively [13]. These findings raise interesting questions regarding the biological and genetic species concepts in ticks belonging to the genus *Rhipicephalus*. The ability of ticks from different species to mate and generate fertile hybrids has been previously demonstrated in the laboratory, for instance, with *Rh. appendiculatus* and *Rh. zambeziensis* [34]. Altogether, these data suggest that the results of crossbreeding experiments and phylogenetic analysis may not be concordant and therefore should be carefully interpreted while assessing the conspecificity or distinctiveness of closely related species belonging to this genus.

Other researchers have recognized that *Rhipicephalus* sp. I and *Rhipicephalus* sp. II are different evolutionary entities [19, 35]. Indeed, recent studies indicated that the distribution of these two temperate lineages is disrupted, with *Rhipicephalus* sp. I being found in Africa (north of the Sahara) and south-eastern Europe, and *Rhipicephalus* sp. II being predominantly found from the middle to the western part of Europe [19, 23, 35]. Interestingly, both lineages have been found in Italy, with *Rhipicephalus* sp. I reported in the south (Puglia and Sicily) and *Rhipicephalus* sp. II in both the south (Sicily) and the north (Verona) [13, 21]. This suggests that these lineages may occur in sympatry in southern Italy, but probably in a limited geographical area. However, their actual distribution ranges across the country and the possible areas of sympatry remain to be investigated. In the same way, the driving factors for their genetic differentiation and apparent incomplete reproductive isolation are unknown. Factors such as temporal (e.g. seasonal shift) and spatial isolation (e.g. habitat preference) may not be enough to explain these differences as both lineages studied herein display similar seasonal patterns and are predominately parasitic on dogs [23, 36, 37].

The occurrence of hybrids in sympatric zones as well as their impact (if any) in the occurrence of certain pathogens should be investigated. For instance, a study evaluated the vector capacity of ticks from four populations (i.e. two from Brazil, one from Argentina and one from Uruguay) of *Rh. sanguineus* (*s.l.*) for transmitting *E. canis* [20]. The study showed that only ticks from a population from south-eastern Brazil (belonging to the tropical lineage) were able to transmitting the bacterium to naïve dogs. Further research is needed to assess the vectorial competence of *Rhipicephalus* sp. I and *Rhipicephalus* sp. II for human pathogens, including the bacterium *R. conorii*, the main causative agent of Mediterranean spotted fever.

Both the pure line of *Rhipicephalus* sp. II and the cross between *Rhipicephalus* sp. II females and *Rhipicephalus* sp. I males generated larvae, nymphs and adults presenting the same mtDNA genotype of their female progenitor. On the other hand, the cross between *Rhipicephalus* sp. I females and *Rhipicephalus* sp. II males generated larvae and nymphs presenting either *Rhipicephalus* sp. I or *Rhipicephalus* sp. II mtDNA genotypes. This suggests the occurrence of paternal leakage (i.e. transmission of mitochondrial DNA from father to offspring) or mitochondrial heteroplasmy of parental females (i.e. presence of multiple mitochondrial genotypes within an individual). This hypothesis opens up new research avenues concerning mitochondrial inheritance and heteroplasmy in ticks and should be investigated in future studies.

Interestingly, adult ticks from all groups presented mtDNA of their mothers. The finding of paternal mtDNA in larvae and nymphs and the absence in adults descending from *Rhipicephalus* sp. I females may suggest that the persistence of paternal mtDNA or heteroplasmy may vary across tick developmental stages. For instance, it has been shown that heteroplasmy frequency changes between tissues of the same individual and between generations in humans [38]. It is also worth mentioning that the detection of heteroplasmy by DNA sequencing is challenging if one of the haplotypes occurs at low frequency [39]. These hypotheses should be investigated in future large-scale studies with natural populations of these tick lineages.

## Conclusions

The temperate lineages of *Rh. sanguineus* (*s.l.*) studied herein are biologically compatible and genetic data obtained from both pure and hybrid lines suggest the occurrence of paternal inheritance or mitochondrial heteroplasmy. This study opens new research avenues and raises question regarding the usefulness of genetic data and crossbreeding experiments as criteria for the definition of cryptic species in ticks.

## Additional files

Additional file 1: **Table S1**. Group, stage, generation and genotype of ticks genetically identified in this study. (DOCX 18 kb)

Additional file 2: Partial cytochrome *c* oxidase subunit 1 (*cox*1) gene sequences generated in this study. (FAS 60 kb)

## Abbreviations

*cox*1 gene: Cytochrome *c* oxidase subunit 1 gene
*crt* gene: Calreticulin gene
mtDNA: Mitochondrial DNA
*Rh.*: *Rhipicephalus*
*s.l.*: *sensu lato*
*s.s.*: *sensu stricto*

## References

[1] Dantas-Torres F, Chomel BB, Otranto D (2012). Ticks and tick-borne diseases: a One Health perspective. Trends Parasitol..

[2] Estrada-Peña A (2015). Ticks as vectors: taxonomy, biology and ecology. Rev Sci Tech..

[3] Dantas-Torres F (2015). Climate change, biodiversity, ticks and tick-borne diseases: the butterfly effect. Int J Parasitol Parasites Wildl..

[4] Nava S, Guglielmone AA, Mangold AJ. An overview of systematics and evolution of ticks. Front Biosci (Landmark Ed). 2009;(14):2857–77. 10.2741/3418 19273240

[5] Dantas-Torres F, Otranto D (2015). Further thoughts on the taxonomy and vector role of *Rhipicephalus sanguineus* group ticks. Vet Parasitol.

[6] Nava S, Estrada-Peña A, Petney T, Beati L, Labruna MB, Szabó MP (2015). The taxonomic status of *Rhipicephalus sanguineus* (Latreille, 1806). Vet Parasitol.

[7] Oliveira PR, Bechara GH, Denardi SE, Saito KC, Nunes ET, Szabó MP (2005). Comparison of the external morphology of *Rhipicephalus sanguineus* (Latreille, 1806) (Acari: Ixodidae) ticks from Brazil and Argentina. Vet Parasitol.

[8] Szabó MP, Mangold AJ, João CF, Bechara GH, Guglielmone AA (2005). Biological and DNA evidence of two dissimilar populations of the *Rhipicephalus sanguineus* tick group (Acari: Ixodidae) in South America. Vet Parasitol.

[9] Burlini L, Teixeira KR, Szabó MP, Famadas KM (2010). Molecular dissimilarities of *Rhipicephalus sanguineus* (Acari: Ixodidae) in Brazil and its relation with samples throughout the world: is there a geographical pattern?. Exp Appl Acarol.

[10] Moraes-Filho J, Marcili A, Nieri-Bastos FA, Richtzenhain LJ, Labruna MB (2011). Genetic analysis of ticks belonging to the *Rhipicephalus sanguineus* group in Latin America. Acta Trop.

[11] Levin ML, Studer E, Killmaster L, Zemtsova G, Mumcuoglu KY (2012). Crossbreeding between different geographical populations of the brown dog tick, *Rhipicephalus sanguineus* (Acari: Ixodidae). Exp Appl Acarol.

[12] Nava S, Mastropaolo M, Venzal JM, Mangold AJ, Guglielmone AA (2012). Mitochondrial DNA analysis of *Rhipicephalus sanguineus* sensu lato (Acari: Ixodidae) in the Southern Cone of South America. Vet Parasitol.

[13] Dantas-Torres F, Latrofa MS, Annoscia G, Giannelli A, Parisi A, Otranto D (2013). Morphological and genetic diversity of *Rhipicephalus sanguineus sensu lato* from the New and Old Worlds. Parasit Vectors.

[14] Liu GH, Chen F, Chen YZ, Song HQ, Lin RQ, Zhou DH (2013). Complete mitochondrial genome sequence data provides genetic evidence that the brown dog tick *Rhipicephalus sanguineus* (Acari: Ixodidae) represents a species complex. Int J Biol Sci.

[15] Hekimoğlu O, Sağlam İK, Özer N, Estrada-Peña A (2016). New molecular data shed light on the global phylogeny and species limits of the *Rhipicephalus sanguineus* complex. Ticks Tick Borne Dis.

[16] Sanches GS, Évora PM, Mangold AJ, Jittapalapong S, Rodriguez-Mallon A, Guzmán PE (2016). Molecular, biological, and morphometric comparisons between different geographical populations of *Rhipicephalus sanguineus sensu lato* (Acari: Ixodidae). Vet Parasitol.

[17] Zemtsova GE, Apanaskevich DA, Reeves WK, Hahn M, Snellgrove A, Levin ML (2016). Phylogeography of *Rhipicephalus sanguineus sensu lato* and its relationships with climatic factors. Exp Appl Acarol.

[18] Caetano RL, Vizzoni VF, Bitencourth K, Carriço C, Sato TP, Pinto ZT (2017). Ultrastructural morphology and molecular analyses of tropical and temperate “species” of *Rhipicephalus sanguineus sensu lato* (Acari: Ixodidae) in Brazil. J Med Entomol.

[19] Hornok S, Sándor AD, Tomanović S, Beck R, D'Amico G, Kontschán J (2017). East and west separation of *Rhipicephalus sanguineus* mitochondrial lineages in the Mediterranean Basin. Parasit Vectors.

[20] Moraes-Filho J, Krawczak FS, Costa FB, Soares JF, Labruna MB (2015). Comparative evaluation of the vector competence of four South American populations of the *Rhipicephalus sanguineus* group for the bacterium *Ehrlichia canis*, the agent of canine monocytic ehrlichiosis. PLoS One.

[21] Latrofa MS, Dantas-Torres F, Giannelli A, Otranto D (2014). Molecular detection of tick-borne pathogens in *Rhipicephalus sanguineus* group ticks. Ticks Tick Borne Dis.

[22] Porretta D, Latrofa MS, Dantas-Torres F, Mastrantonio V, Iatta R, Otranto D (2016). Exon-intron structure and sequence variation of the calreticulin gene among *Rhipicephalus sanguineus* group ticks. Parasit Vectors.

[23] Dantas-Torres F, Maia C, Latrofa MS, Annoscia G, Cardoso L, Otranto D (2017). Genetic characterization of *Rhipicephalus sanguineus* (*sensu lato*) ticks from dogs in Portugal. Parasit Vectors.

[24] Dantas-Torres F, Figueredo LA, Otranto D (2011). Seasonal variation in the effect of climate on the biology of *Rhipicephalus sanguineus* in southern Europe. Parasitology..

[25] Walter DE, Krantz GW, Krantz GW, Walter DE (2009). Collecting, rearing, and preparing specimens. A manual of acarology.

[26] Murrell A, Campbell NJ, Barker SC (2000). Phylogenetic analyses of the rhipicephaline ticks indicate that the genus *Rhipicephalus* is paraphyletic. Mol Phylogenet Evol.

[27] Larkin MA, Blackshields G, Brown NP, Chenna R, McGettigan PA, McWilliam H (2007). Clustal W and Clustal X version 2.0. Bioinformatics.

[28] Filippova NA (1997). Ixodid ticks of subfamily Amblyomminae. Fauna of Russia and neighbouring countries.

[29] Szabó MP, Bertipaglia EC, Bechara GH (2000). Cross reactivity between instars of the *Rhipicephalus sanguineus* (Latreille, 1806) tick. Ann N Y Acad Sci.

[30] Labruna MB, Gerardi M, Krawczak FS, Moraes-Filho J (2017). Comparative biology of the tropical and temperate species of *Rhipicephalus sanguineus sensu lato* (Acari: Ixodidae) under different laboratory conditions. Ticks Tick Borne Dis.

[31] Oliver JH, Wilkinson PR, Kohls GM (1972). Observations on hybridization of three species of North American *Dermacentor* ticks. J Parasitol.

[32] Davey RB, Cooksey LM, Despins JL (1991). Survival of larvae of *Boophilus annulatus*, *Boophilus microplus*, and *Boophilus* hybrids (Acari: Ixodidae) in different temperature and humidity regimes in the laboratory. Vet Parasitol.

[33] Araya-Anchetta A, Scoles GA, Giles J, Busch JD, Wagner DM (2013). Hybridization in natural sympatric populations of *Dermacentor* ticks in northwestern North America. Ecol Evol.

[34] Zivkovic D, Pegram RG, Jongejan F, Mwase ET (1986). Biology of *Rhipicephalus appendiculatus* and *R. zambeziensis* and production of a fertile hybrid under laboratory conditions. Exp Appl Acarol.

[35] Chitimia-Dobler L, Langguth J, Pfeffer M, Kattner S, Küpper T, Friese D (2017). Genetic analysis of *Rhipicephalus sanguineus sensu lato* ticks parasites of dogs in Africa north of the Sahara based on mitochondrial DNA sequences. Vet Parasitol.

[36] Gilot B, Diop S, Laforge ML (1992). Dynamique spatio-temporelle des populations de *Rhipicephalus sanguineus* (Latreille, 1806) (Acariens Ixodoidea) dans una cite HLM de Marseille. Acarologia.

[37] Lorusso V, Dantas-Torres F, Lia RP, Tarallo VD, Mencke N, Capelli G, Otranto D (2010). Seasonal dynamics of the brown dog tick, *Rhipicephalus sanguineus*, on a confined dog population in Italy. Med Vet Entomol.

[38] Rebolledo-Jaramillo B, Su MS, Stoler N, McElhoe JA, Dickins B, Blankenberg D (2014). Maternal age effect and severe germ-line bottleneck in the inheritance of human mitochondrial DNA. Proc Natl Acad Sci USA.

[39] Nunes MD, Dolezal M, Schlötterer C (2013). Extensive paternal mtDNA leakage in natural populations of *Drosophila melanogaster*. Mol Ecol.

