# Supplementary material for: Biological compatibility between two temperate lineages of brown dog ticks, Rhipicephalus sanguineus (sensu lato)
Source: Parasit Vectors. 2018 Jul 9;11:398. doi: 10.1186/s13071-018-2941-2 (PMC6038233; doi:10.1186/s13071-018-2941-2)
Supplement: Supplementary file 1 — Table S1. Group, stage, generation and genotype of ticks genetically identified in this study. (DOCX 18 kb) [file 13071_2018_2941_MOESM1_ESM.docx]

**Table S1** Group, stage, generation and genotype of ticks genetically identified in this study

| **Group** | **Stage** | **Generation** | **Genotype** |
| --- | --- | --- | --- |
| Italy (Putignano) | Male | Wildtype | *Rhipicephalus* sp. I |
| Italy (Putignano) | Male | Wildtype | *Rhipicephalus* sp. I |
| Italy (Putignano) | Female | Wildtype | *Rhipicephalus* sp. I |
| Italy (Putignano) | Female | Wildtype | *Rhipicephalus* sp. I |
| Italy (Putignano) | Larva | Wildtype | *Rhipicephalus* sp. I |
| Italy (Putignano) | Larva | Wildtype | *Rhipicephalus* sp. I |
| Italy (Putignano) | Larva | Wildtype | *Rhipicephalus* sp. I |
| Italy (Putignano) | Larva | Wildtype | *Rhipicephalus* sp. I |
| Italy (Putignano) | Larva | Wildtype | *Rhipicephalus* sp. I |
| Italy (Putignano) | Nymph | Wildtype | *Rhipicephalus* sp. I |
| Italy (Putignano) | Nymph | Wildtype | *Rhipicephalus* sp. I |
| Portugal (Faro) | Male | Wildtype | *Rhipicephalus* sp. II |
| Portugal (Faro) | Male | Wildtype | *Rhipicephalus* sp. II |
| Portugal (Faro) | Female | Wildtype | *Rhipicephalus* sp. II |
| Portugal (Faro) | Female | Wildtype | *Rhipicephalus* sp. II |
| Portugal (Faro) | Larva | Wildtype | *Rhipicephalus* sp. II |
| Portugal (Faro) | Larva | Wildtype | *Rhipicephalus* sp. II |
| Portugal (Faro) | Larva | Wildtype | *Rhipicephalus* sp. II |
| Portugal (Faro) | Nymph | Wildtype | *Rhipicephalus* sp. II |
| Portugal (Faro) | Nymph | Wildtype | *Rhipicephalus* sp. II |
| Portugal (Faro) | Nymph | Wildtype | *Rhipicephalus* sp. II |
| G1 (*Rhipicephalus* sp. II female / *Rhipicephalus* sp. II male) | Larva | F1 | *Rhipicephalus* sp. II |
| G1 (*Rhipicephalus* sp. II female / *Rhipicephalus* sp. II male) | Larva | F1 | *Rhipicephalus* sp. II |
| G1 (*Rhipicephalus* sp. II female / *Rhipicephalus* sp. II male) | Larva | F1 | *Rhipicephalus* sp. II |
| G1 (*Rhipicephalus* sp. II female / *Rhipicephalus* sp. II male) | Larva | F1 | *Rhipicephalus* sp. II |
| G1 (*Rhipicephalus* sp. II female / *Rhipicephalus* sp. II male) | Larva | F1 | *Rhipicephalus* sp. II |
| G1 (*Rhipicephalus* sp. II female / *Rhipicephalus* sp. II male) | Nymph | F1 | *Rhipicephalus* sp. II |
| G1 (*Rhipicephalus* sp. II female / *Rhipicephalus* sp. II male) | Nymph | F1 | *Rhipicephalus* sp. II |
| G1 (*Rhipicephalus* sp. II female / *Rhipicephalus* sp. II male) | Nymph | F1 | *Rhipicephalus* sp. II |
| G1 (*Rhipicephalus* sp. II female / *Rhipicephalus* sp. II male) | Male | F1 | *Rhipicephalus* sp. II |
| G1 (*Rhipicephalus* sp. II female / *Rhipicephalus* sp. II male) | Male | F1 | *Rhipicephalus* sp. II |
| G1 (*Rhipicephalus* sp. II female / *Rhipicephalus* sp. II male) | Female | F1 | *Rhipicephalus* sp. II |
| G1 (*Rhipicephalus* sp. II female / *Rhipicephalus* sp. II male) | Female | F1 | *Rhipicephalus* sp. II |
| G2 (*Rhipicephalus* sp. I female / *Rhipicephalus* sp. I male) | Larva | F1 | *Rhipicephalus* sp. II |
| G2 (*Rhipicephalus* sp. I female / *Rhipicephalus* sp. I male) | Nymph | F1 | *Rhipicephalus* sp. I |
| G2 (*Rhipicephalus* sp. I female / *Rhipicephalus* sp. I male) | Nymph | F1 | *Rhipicephalus* sp. I |
| G2 (*Rhipicephalus* sp. I female / *Rhipicephalus* sp. I male) | Nymph | F1 | *Rhipicephalus* sp. I |
| G2 (*Rhipicephalus* sp. I female / *Rhipicephalus* sp. I male) | Nymph | F1 | *Rhipicephalus* sp. I |
| G2 (*Rhipicephalus* sp. I female / *Rhipicephalus* sp. I male) | Nymph | F1 | *Rhipicephalus* sp. I |
| G2 (*Rhipicephalus* sp. I female / *Rhipicephalus* sp. I male) | Nymph | F1 | *Rhipicephalus* sp. II |
| G2 (*Rhipicephalus* sp. I female / *Rhipicephalus* sp. I male) | Male | F1 | *Rhipicephalus* sp. I |
| G2 (*Rhipicephalus* sp. I female / *Rhipicephalus* sp. I male) | Male | F1 | *Rhipicephalus* sp. I |
| G2 (*Rhipicephalus* sp. I female / *Rhipicephalus* sp. I male) | Male | F1 | *Rhipicephalus* sp. I |
| G2 (*Rhipicephalus* sp. I female / *Rhipicephalus* sp. I male) | Male | F1 | *Rhipicephalus* sp. I |
| G2 (*Rhipicephalus* sp. I female / *Rhipicephalus* sp. I male) | Male | F1 | *Rhipicephalus* sp. I |
| G2 (*Rhipicephalus* sp. I female / *Rhipicephalus* sp. I male) | Male | F1 | *Rhipicephalus* sp. I |
| G2 (*Rhipicephalus* sp. I female / *Rhipicephalus* sp. I male) | Male | F1 | *Rhipicephalus* sp. I |
| G2 (*Rhipicephalus* sp. I female / *Rhipicephalus* sp. I male) | Male | F1 | *Rhipicephalus* sp. I |
| G2 (*Rhipicephalus* sp. I female / *Rhipicephalus* sp. I male) | Male | F1 | *Rhipicephalus* sp. I |
| G2 (*Rhipicephalus* sp. I female / *Rhipicephalus* sp. I male) | Male | F1 | *Rhipicephalus* sp. I |
| G2 (*Rhipicephalus* sp. I female / *Rhipicephalus* sp. I male) | Female | F1 | *Rhipicephalus* sp. I |
| G2 (*Rhipicephalus* sp. I female / *Rhipicephalus* sp. I male) | Female | F1 | *Rhipicephalus* sp. I |
| G2 (*Rhipicephalus* sp. I female / *Rhipicephalus* sp. I male) | Female | F1 | *Rhipicephalus* sp. I |
| G2 (*Rhipicephalus* sp. I female / *Rhipicephalus* sp. I male) | Female | F1 | *Rhipicephalus* sp. I |
| G2 (*Rhipicephalus* sp. I female / *Rhipicephalus* sp. I male) | Female | F1 | *Rhipicephalus* sp. I |
| G2 (*Rhipicephalus* sp. I female / *Rhipicephalus* sp. I male) | Female | F1 | *Rhipicephalus* sp. I |
| G2 (*Rhipicephalus* sp. I female / *Rhipicephalus* sp. I male) | Female | F1 | *Rhipicephalus* sp. I |
| G2 (*Rhipicephalus* sp. I female / *Rhipicephalus* sp. I male) | Female | F1 | *Rhipicephalus* sp. I |
| G2 (*Rhipicephalus* sp. I female / *Rhipicephalus* sp. I male) | Female | F1 | *Rhipicephalus* sp. I |
| G2 (*Rhipicephalus* sp. I female / *Rhipicephalus* sp. I male) | Female | F1 | *Rhipicephalus* sp. I |
| G3 (*Rhipicephalus* sp. II female / *Rhipicephalus* sp. I male) | Larva | F1 | *Rhipicephalus* sp. II |
| G3 (*Rhipicephalus* sp. II female / *Rhipicephalus* sp. I male) | Larva | F1 | *Rhipicephalus* sp. II |
| G3 (*Rhipicephalus* sp. II female / *Rhipicephalus* sp. I male) | Larva | F1 | *Rhipicephalus* sp. II |
| G3 (*Rhipicephalus* sp. II female / *Rhipicephalus* sp. I male) | Larva | F1 | *Rhipicephalus* sp. II |
| G3 (*Rhipicephalus* sp. II female / *Rhipicephalus* sp. I male) | Larva | F1 | *Rhipicephalus* sp. II |
| G3 (*Rhipicephalus* sp. II female / *Rhipicephalus* sp. I male) | Larva | F1 | *Rhipicephalus* sp. II |
| G3 (*Rhipicephalus* sp. II female / *Rhipicephalus* sp. I male) | Larva | F1 | *Rhipicephalus* sp. II |
| G3 (*Rhipicephalus* sp. II female / *Rhipicephalus* sp. I male) | Larva | F1 | *Rhipicephalus* sp. II |
| G3 (*Rhipicephalus* sp. II female / *Rhipicephalus* sp. I male) | Larva | F1 | *Rhipicephalus* sp. II |
| G3 (*Rhipicephalus* sp. II female / *Rhipicephalus* sp. I male) | Larva | F1 | *Rhipicephalus* sp. II |
| G3 (*Rhipicephalus* sp. II female / *Rhipicephalus* sp. I male) | Nymph | F1 | *Rhipicephalus* sp. II |
| G3 (*Rhipicephalus* sp. II female / *Rhipicephalus* sp. I male) | Nymph | F1 | *Rhipicephalus* sp. II |
| G3 (*Rhipicephalus* sp. II female / *Rhipicephalus* sp. I male) | Nymph | F1 | *Rhipicephalus* sp. II |
| G3 (*Rhipicephalus* sp. II female / *Rhipicephalus* sp. I male) | Nymph | F1 | *Rhipicephalus* sp. II |
| G3 (*Rhipicephalus* sp. II female / *Rhipicephalus* sp. I male) | Nymph | F1 | *Rhipicephalus* sp. II |
| G3 (*Rhipicephalus* sp. II female / *Rhipicephalus* sp. I male) | Nymph | F1 | *Rhipicephalus* sp. II |
| G3 (*Rhipicephalus* sp. II female / *Rhipicephalus* sp. I male) | Nymph | F1 | *Rhipicephalus* sp. II |
| G3 (*Rhipicephalus* sp. II female / *Rhipicephalus* sp. I male) | Nymph | F1 | *Rhipicephalus* sp. II |
| G3 (*Rhipicephalus* sp. II female / *Rhipicephalus* sp. I male) | Nymph | F1 | *Rhipicephalus* sp. II |
| G3 (*Rhipicephalus* sp. II female / *Rhipicephalus* sp. I male) | Nymph | F1 | *Rhipicephalus* sp. II |
| G3 (*Rhipicephalus* sp. II female / *Rhipicephalus* sp. I male) | Male | F1 | *Rhipicephalus* sp. II |
| G3 (*Rhipicephalus* sp. II female / *Rhipicephalus* sp. I male) | Male | F1 | *Rhipicephalus* sp. II |
| G3 (*Rhipicephalus* sp. II female / *Rhipicephalus* sp. I male) | Male | F1 | *Rhipicephalus* sp. II |
| G3 (*Rhipicephalus* sp. II female / *Rhipicephalus* sp. I male) | Male | F1 | *Rhipicephalus* sp. II |
| G3 (*Rhipicephalus* sp. II female / *Rhipicephalus* sp. I male) | Male | F1 | *Rhipicephalus* sp. II |
| G3 (*Rhipicephalus* sp. II female / *Rhipicephalus* sp. I male) | Male | F1 | *Rhipicephalus* sp. II |
| G3 (*Rhipicephalus* sp. II female / *Rhipicephalus* sp. I male) | Male | F1 | *Rhipicephalus* sp. II |
| G3 (*Rhipicephalus* sp. II female / *Rhipicephalus* sp. I male) | Male | F1 | *Rhipicephalus* sp. II |
| G3 (*Rhipicephalus* sp. II female / *Rhipicephalus* sp. I male) | Male | F1 | *Rhipicephalus* sp. II |
| G3 (*Rhipicephalus* sp. II female / *Rhipicephalus* sp. I male) | Female | F1 | *Rhipicephalus* sp. II |
| G3 (*Rhipicephalus* sp. II female / *Rhipicephalus* sp. I male) | Female | F1 | *Rhipicephalus* sp. II |
| G3 (*Rhipicephalus* sp. II female / *Rhipicephalus* sp. I male) | Female | F1 | *Rhipicephalus* sp. II |
| G3 (*Rhipicephalus* sp. II female / *Rhipicephalus* sp. I male) | Female | F1 | *Rhipicephalus* sp. II |
| G3 (*Rhipicephalus* sp. II female / *Rhipicephalus* sp. I male) | Female | F1 | *Rhipicephalus* sp. II |
| G3 (*Rhipicephalus* sp. II female / *Rhipicephalus* sp. I male) | Female | F1 | *Rhipicephalus* sp. II |
| G3 (*Rhipicephalus* sp. II female / *Rhipicephalus* sp. I male) | Female | F1 | *Rhipicephalus* sp. II |
| G3 (*Rhipicephalus* sp. II female / *Rhipicephalus* sp. I male) | Female | F1 | *Rhipicephalus* sp. II |
| G3 (*Rhipicephalus* sp. II female / *Rhipicephalus* sp. I male) | Female | F1 | *Rhipicephalus* sp. II |
| G3 (*Rhipicephalus* sp. II female / *Rhipicephalus* sp. I male) | Female | F1 | *Rhipicephalus* sp. II |
| G4 (*Rhipicephalus* sp. I female / *Rhipicephalus* sp. II male) | Larva | F1 | *Rhipicephalus* sp. II |
| G4 (*Rhipicephalus* sp. I female / *Rhipicephalus* sp. II male) | Larva | F1 | *Rhipicephalus* sp. II |
| G4 (*Rhipicephalus* sp. I female / *Rhipicephalus* sp. II male) | Larva | F1 | *Rhipicephalus* sp. II |
| G4 (*Rhipicephalus* sp. I female / *Rhipicephalus* sp. II male) | Larva | F1 | *Rhipicephalus* sp. II |
| G4 (*Rhipicephalus* sp. I female / *Rhipicephalus* sp. II male) | Larva | F1 | *Rhipicephalus* sp. II |
| G4 (*Rhipicephalus* sp. I female / *Rhipicephalus* sp. II male) | Larva | F1 | *Rhipicephalus* sp. II |
| G4 (*Rhipicephalus* sp. I female / *Rhipicephalus* sp. II male) | Larva | F1 | *Rhipicephalus* sp. I |
| G4 (*Rhipicephalus* sp. I female / *Rhipicephalus* sp. II male) | Nymph | F1 | *Rhipicephalus* sp. I |
| G4 (*Rhipicephalus* sp. I female / *Rhipicephalus* sp. II male) | Nymph | F1 | *Rhipicephalus* sp. I |
| G4 (*Rhipicephalus* sp. I female / *Rhipicephalus* sp. II male) | Nymph | F1 | *Rhipicephalus* sp. I |
| G4 (*Rhipicephalus* sp. I female / *Rhipicephalus* sp. II male) | Male | F1 | *Rhipicephalus* sp. I |
| G4 (*Rhipicephalus* sp. I female / *Rhipicephalus* sp. II male) | Male | F1 | *Rhipicephalus* sp. I |
| G4 (*Rhipicephalus* sp. I female / *Rhipicephalus* sp. II male) | Male | F1 | *Rhipicephalus* sp. I |
| G4 (*Rhipicephalus* sp. I female / *Rhipicephalus* sp. II male) | Male | F1 | *Rhipicephalus* sp. I |
| G4 (*Rhipicephalus* sp. I female / *Rhipicephalus* sp. II male) | Male | F1 | *Rhipicephalus* sp. I |
| G4 (*Rhipicephalus* sp. I female / *Rhipicephalus* sp. II male) | Female | F1 | *Rhipicephalus* sp. I |
| G4 (*Rhipicephalus* sp. I female / *Rhipicephalus* sp. II male) | Female | F1 | *Rhipicephalus* sp. I |
| G4 (*Rhipicephalus* sp. I female / *Rhipicephalus* sp. II male) | Female | F1 | *Rhipicephalus* sp. I |
| G4 (*Rhipicephalus* sp. I female / *Rhipicephalus* sp. II male) | Female | F1 | *Rhipicephalus* sp. I |
| G4 (*Rhipicephalus* sp. I female / *Rhipicephalus* sp. II male) | Female | F1 | *Rhipicephalus* sp. I |
| G4 (*Rhipicephalus* sp. I female / *Rhipicephalus* sp. II male) | Female | F1 | *Rhipicephalus* sp. I |
| G4 (*Rhipicephalus* sp. I female / *Rhipicephalus* sp. II male) | Female | F1 | *Rhipicephalus* sp. I |
| G4 (*Rhipicephalus* sp. I female / *Rhipicephalus* sp. II male) | Female | F1 | *Rhipicephalus* sp. I |
